# Assessing responsiveness of the EQ-5D-3L, the Oxford Hip Score, and the Oxford Knee Score in the NHS patient-reported outcome measures

**DOI:** 10.1186/s13018-020-02126-2

**Published:** 2021-01-07

**Authors:** Sujin Kang

**Affiliations:** Faculty of Medicine, Imperial College London, South Kensington Campus, London, SW7 2AZ UK

**Keywords:** Hip and knee replacement, Patient-reported outcome, EQ-5D-3L Index, OHS, OKS, Internal responsiveness

## Abstract

**Background:**

The degree to which a validated instrument is able to detect clinically significant change over time is an important issue for the better management of hip or knee replacement surgery. This study examines the internal responsiveness of the EQ-5D-3L, the Oxford Hip Score (OHS), and the Oxford Knee Score (OKS) by various methods. Data from NHS patient-reported outcome measures (PROMs) linked to the Hospital Episodes Statistics (HES) dataset (2009–2015) was analysed for patients who underwent primary hip surgery (*N* = 181,424) and primary knee surgery (*N* = 191,379).

**Methods:**

Paired data-specific univariate responsiveness was investigated using the standardized response mean (SRM), the standardized effect size (SES), and the responsiveness index (RI). Multivariate responsiveness was furthermore examined using the defined capacity of benefit score (i.e. paired data-specific MCID), adjusting baseline covariates such as age, gender, and comorbidities in the Box-Cox regression models. The observed and predicted percentages of patient improvement were examined both as a whole and by the patients' self-assessed transition level.

**Results:**

The results showed that both the OHS and the OKS demonstrated great univariate and multivariate responsiveness. The percentages of the observed (predicted) total improvement were high: 51 (54)% in the OHS and 73 (58)% in OKS. The OHS and the OKS showed distinctive differences in improvement by the 3-level transition, i.e. *a little better* vs. *about the same* vs. *a little worse*. The univariate responsiveness of the EQ-5D-3L showed moderate effects in total by Cohen’s thresholds. The percentages of improvement in the EQ-5D-3L were moderate: 44 (48)% in the hip and 42 (44)% for the knee replacement population.

**Conclusions:**

Distinctive percentage differences in patients’ perception of improvement were observed when the paired data-specific capacity of benefit score was applied to examine responsiveness. This is useful in clinical practice as rationale for access to surgery at the individual-patient level. This study shows the importance of analytic methods and instruments for investigation of the health status in hip and/or knee replacement surgery. The study finding also supports the idea of using a generic measure along with the disease-specific instruments in terms of cross-validation.

## Background

The responsiveness of a health-related functional state is an important issue in arthroplasty surgery. Responsiveness is the ability of an instrument to detect clinically significant change in health status and as such reflects its impact on clinical practice over time [1–3]. It is well recognized that measurement properties can vary according to the study population of interest. This is particularly true of the generic measures, especially those measuring responsiveness. The decision to use a generic or disease-specific instrument to detect responsiveness will also depend on the study design, objectives, and evaluation of cost-effectiveness [4]. Generic health status measures seek a broad perspective that is not specifically related to the restricted scope of the health-related functional status of a particular disease. Generic measures allow the comparison of health status across different diseases and interventions [4, 5].

Assessing outcomes of hip and knee replacement surgery for both generic and specific measures is enabled by the EQ-5D-3L, the Oxford Hip Score (OHS), and the Oxford Knee Score (OKS). The EQ-5D is a well-known and widely used generic patient-reported outcome questionnaire [6]. The current UK version of EQ-5D-3L was introduced in 1997 as a generic measure of health for clinical and economic assessment [7]. It was designed to describe and value health by providing a single summary index-based value (utility; − 0.59 to 1) representing the overall health-related quality of life by quantifying a preference for the individual’s health state [8]. The questionnaire consists of a self-reported/descriptive system to describe the three-level health problems (no/some/extreme problem) on each five dimension: mobility (i.e. problem in walking), self-care (i.e. problem with self-care), usual activities (e.g. work, study, housework, family, or leisure activities), pain/discomfort, and anxiety/depression.

The OHS and the OKS focus on the disease being studied, allowing greater sensitivity to intervention related-change compared to generic measures [4, 9]. The OHS and the OKS consist of 12 Likert-type response items, which relate to pain and disability experienced over the past 4 weeks [10, 11]. Scores from each item are summed (responses coded from ‘None’ = 4 to ‘Severe’ = 0) providing a range of 0 to 48, with a higher score indicating greater health status [12, 13].

Husted et al. [14] defined the internal responsiveness as the ability of a measure to change over a pre-specified time frame. The external responsiveness was defined as the extent to which changes in measure relate to changes in other measures of health status, and it measures rather the relationship between change in the measure and change in the external standard [14]. The external responsiveness between independent groups and cross-validation between measuring systems were explored in the previous studies. The ability of these instruments to detect responsiveness is required to examine using the paired group specific statistics as previous studies did not specify the internal responsiveness for a single group. The aim of this study is to evaluate the paired data-specific responsiveness of the EQ-5D-3L, the OHS, and the OKS using various analytic methods, and to discuss which analytic methods and instruments should be used for the reporting system in arthroplasty surgery.

## Methods

### Data sources

Responsiveness was accessed for the population from the NHS patient-reported outcome measures (PROMs) data who have undergone hip or knee surgery in the UK (ref: NIC-392690-F7H2Q). Follow-up was measured 6 months after the hip or knee surgeries. The NHS PROMs linked to Hospital Episodes Statistics (HES) (2009–2015) data recorded the pre- and the 6 months post-operative PROMs outcomes. The outcomes include the EQ-5D-3L and the respective hip and knee Oxford scores for all individuals who underwent hip and knee surgery in England [15].

The inclusion criteria were patients who had not received revision (primary surgery only)^1^ and who had not had previous surgery, using the ‘*Q1_PREVIOUS_SURGERY*’ question (*N* = 575,980). In addition, patients who completed both pre- and post-questionnaires were included, using the ‘Q1 and Q2 Complete’ questions (*N* = 443,262) [16]. For hip surgery, patients who submitted specific data were included for both the pre- and post-operative Oxford questionnaires to derive scores with sufficient procedure, using the ‘HR Q1 and HR Q2 Score Complete’ questions (*N* = 209,761) and the ‘Q1 and Q2 EQ-5D Health Scale Complete Indicators’ (*N* = 181,424). The same approach was applied for those undergoing knee surgeries (*N* = 191,379).

### Outcome and predictor variables

The change scores (the difference between the post- and pre-operative scores) of the EQ-5D-3L, the OHS and the OKS were used as the main outcomes, respectively. The pre-operative EQ-5D-3L, OHS, and OKS scores were used as the main predictor variables for the change scores. Patients' age, gender and important clinical exposures, namely, 12 individual comorbidities (heart disease, high blood pressure, stroke, circulation, lung disease, diabetes, kidney, nervous system, liver disease, cancer, depression, arthritis) were used as other prognostic variables.

### Transition question

The MCID (minimally clinically important difference), which can be linked to the improvement concept, was calculated using the patients’ self-assessment of the 6 months post-operative outcomes relative to the pre-operation. The MCID allows an estimation of the probability of a relevant improvement in the instrument of an intervention [17]. The assumption of the MCID is that the mean change score needed to obtain a medium or large effect size is clinically meaningful [18]. Clinically meaningful refers to a change that indicates the efficacy of the intervention in domains of a health-related functional status instrument [4]. The MCID can be calculated for the group reflecting level (using the anchor-based transition in which the concept of ‘minimal importance’ is explicitly incorporated) and also for the distribution-based individual level (using the standardized response mean (SRM) applied paired data-specific MCID) [17, 19, 20]. In this paper, a combined approach, firstly, the SRM applied paired data-specific MCID was used to estimate the threshold for improvement, and secondly, patients’ perception of improvement was estimated by the level of the transition in the multivariate regression models (Table 1).

**Table 1:** The patient-reported success of the surgery in the Oxford hip (or knee) score questionnaire

Question: Overall, how the problems now in the hip (or knee) on which you had surgery, compared to before operation? Answer: *Much better*—*A little better*—*About the same*—*A little worse*—*Much worse*	

The NHS PROMs contains the post-operative satisfaction and success questions, and the success question was applied in this study since it is considered more objective than the satisfaction question asking ‘How would you describe the results of your operation? Excellent/Very good/Good/Fair/Poor’.

For the paired data-specific univariate responsiveness, the SRM, the standardized effect size (SES), and the responsiveness index (RI) were calculated.

### Univariate responsiveness measures

In the present study, internal responsiveness was investigated focusing on internal standard of an individual using the pre- and post-operation (paired) data and compared as the psychometric property of the EQ-5D-3L, the OHS, and the OKS. The internal responsiveness was assessed by calculating different formula of responsiveness in terms of a critical assessment: the SRM, the SES, and the RI for the univariate statistics.

#### SRM for the paired data [4, 20–22]

1 $$ \mathrm{The}\ \mathrm{paired}\ \mathrm{data}-\mathrm{specific}\ \mathrm{SRM}:\frac{\left({\mathrm{Mean}}_{\mathrm{change}\ \mathrm{score}}/{\mathrm{SD}}_{\mathrm{change}\ \mathrm{score}}\right)}{\surd 2\times \surd \left(1-r\right)} $$

where *r* is a correlation coefficient between the pre- and post-operative scores [4].

The pre- and post-operation data-specific SRM is the ratio between the mean change score and the variability (SD) of that change score within the same group (Mean_change score_/SD_change score_), and the difference between means for the independent data is standardized (i.e. divided) by a value √2 ×  √ (1 − *r*) (as large as would be the case were they independent) [4, 21] (The SRM for the independent data is simply Mean_change score_/SD_change score_ between the two groups [20]).

#### SES for the paired data

The SES was calculated using the patients’ self-assessed transition level, i.e. *much better*, *a little better*, *about the same*, *a little worse*, and *much worse* [4].

2 $$ \mathrm{Standardized}\ \mathrm{Effect}\ \mathrm{Size}\ \left(\mathrm{SES}\right)=\frac{{\mathrm{Mean}}_{\mathrm{pre}-\mathrm{op}.\mathrm{score}}-{\mathrm{Mean}}_{\mathrm{post}-\mathrm{op}.\mathrm{score}\ \left(\mathrm{of}\ \mathrm{the}\ \mathrm{success}\ \mathrm{level}\right)}}{{\mathrm{SD}}_{\mathrm{pre}-\mathrm{op}.\mathrm{score}\ \left(\mathrm{of}\ \mathrm{the}\ \mathrm{success}\ \mathrm{level}\right)}} $$

#### RI for the paired data

The RI was proposed as the ratio of average change produced by a treatment to the between subject variability of difference scores in stable subjects [23]. The RI was calculated using the patients’ self-assessed transition-based (i.e. *a little better* vs. *about the same*) MCID, assuming the patients’ perception of change over time is meaningful [4, 24].

3 $$ \mathrm{Responsiveness}\ \mathrm{Index}\ \left(\mathrm{RI}\right)=\frac{{\mathrm{MCID}}_{\mathrm{anchor}-\mathrm{based}}}{{\mathrm{SD}}_{\mathrm{change}\ \mathrm{score}\ \left(\mathrm{of}\ \mathrm{the}\ \mathrm{stable}\ \mathrm{level}\right)}} $$

where the MCID here is according to a criterion (i.e. the difference in change score between those who perceived *a little better* vs. *about the same*)

In addition to the univariate responsiveness measures, the patients’ perception of improvement was estimated using the modelling approach using the Box-Cox regressions based on log-likelihood while adjusting responsiveness with patient characteristics, including age, gender, and 12 individual comorbidities. For the robust analytic approach, the paired data-specific MCID was defined as the threshold for improvement in the models.

### Multivariate responsiveness measures

#### The threshold for improvement with the MCID for the paired data

Cohen introduced the matched pairs effect size [21], which was later renamed the standardized response mean (SRM) by Liang et al. [4, 20].

The paired data-specific MCID (i.e. Mean_change score_) applied the SRM [Eq. 1], as a desired effect size [25]:

4 $$ \mathrm{The}\ \mathrm{paired}\ \mathrm{data}-\mathrm{specific}\ \mathrm{SRM}\ \left[\mathrm{Eq}\ .1\right]\times \surd 2\times \surd \left(1-r\right)\times {\mathrm{SD}}_{\mathrm{change}\ \mathrm{score}} $$

The independent data MCID, using Cohen’s medium (0.5) or large (0.8) effect size for the independent samples, is Cohen’s *d* (i.e. 0.5 or 0.8) *×* √2 ×  √ (1 − *r*) *×* SD_change score_*.*

#### Multivariate responsiveness using the regression models

**Fig. 1:**
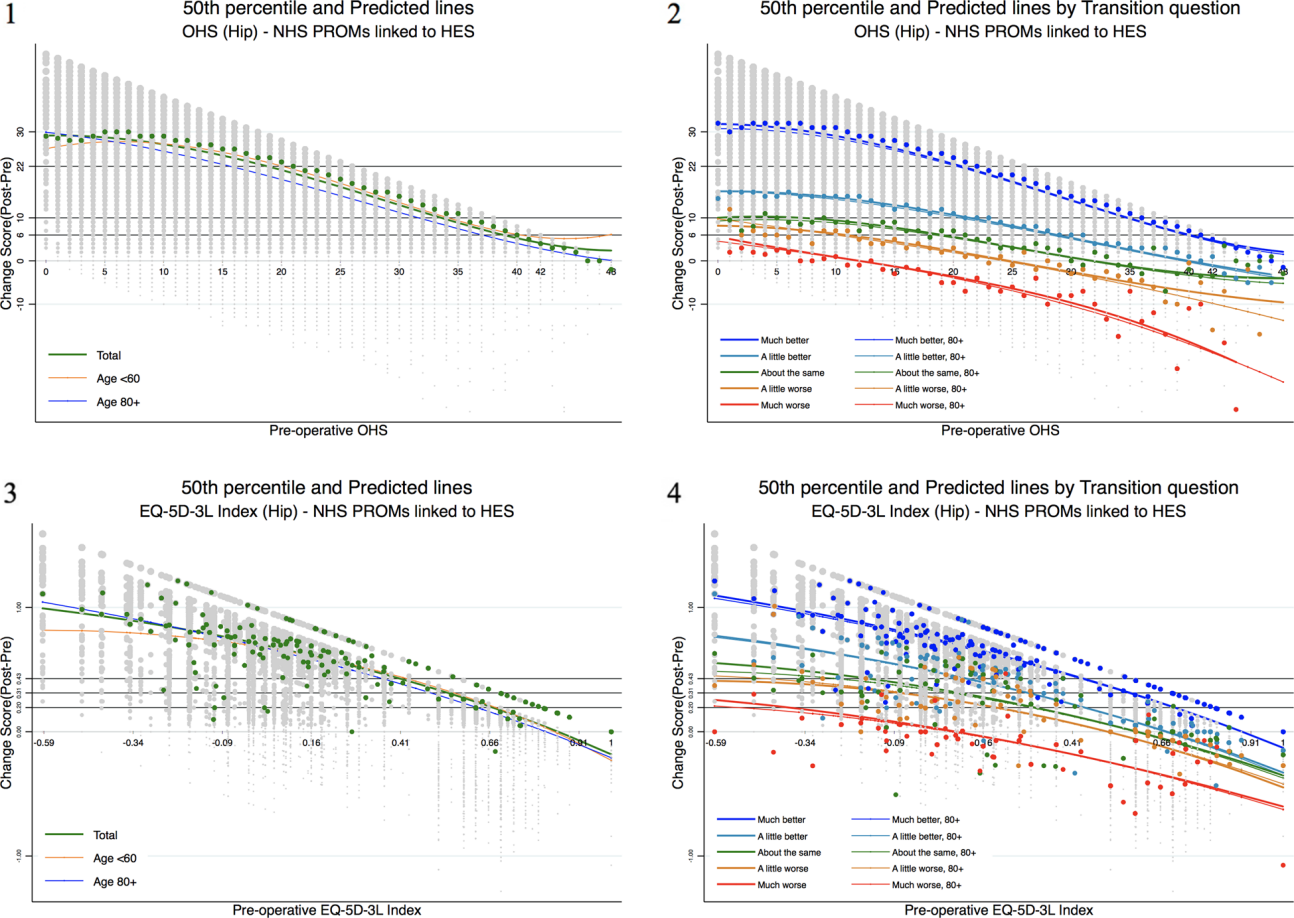
The OHS and EQ-5D-3L – total population (**1**, **3**) and the transition level (**2**, **4**). Fitted 3rd degree Box-Cox regression lines **1** for the OHS total population and **2** by the patients’ self-assessed transition level. The 2nd degree Box-Cox regression estimates **3** for the EQ-5D-3L total hip surgery population and **4** by the patients’ self-assessed transition level. All the graphs are presented by age group additionally. Colourful dots indicate 50th percentile for each category, and grey dots indicate actual observations. Grey horizontal lines indicate each defined score improvement (e.g. 22 for the OHS and 0.428 for the hip EQ-5D-3L). Percentiles of the EQ-5D-3L show all over disperse patterns by the transition level whereas percentiles of the OHS show disperse patterns in ‘A little worse’ and ‘Much worse’ transition level. Model performance of the OKS and the knee EQ-5D-3L is provided in Supplementary Figure 1

The percentage improvement based on the paired data-specific MCID [Eq. 4] was examined as multivariate responsiveness of the EQ-5D-3L, the OHS and the OKS to examine which instrument is sensitive to detect the changes of improvement for the paired data. The result was additionally examined by the patients' self-assessed transition level, i.e. *much better*, *a little better*, *about the same*, *a little worse*, and *much worse*. The observed and estimated percentage improvements were examined separately where regression approaches were applied, adjusting patient baseline covariates such as age, gender, and comorbidities. Adjusting the covariates is one of the strengths in comparison to the responsiveness statistics described in the previous sections. The 3rd and the 2nd degree Box-Cox regressions based on log-likelihood were fitted to estimate the patients’ perception of improvement. The impact of baseline covariates, i.e. age (as a continuous variable), gender, and individual comorbidities, were examined in total and by the transition level population (Fig. 1).

The Box-Cox regression models were selected among other statistical average models (e.g. polynomial regressions) and median-based models (e.g. quantile regressions), after the model diagnostic assessments. The model is robust for a non-normal dependent variable, transforming it into a normal shape. The observational and estimated percentage improvements for the average population were calculated to examine if the instrument has a good discriminative ability. The individual level post-operative scores were modelled as a function of the transformed variables pre-operative linear, quadratic, and cubic terms and of the untransformed age, gender, and individual comorbidities. In comparison to the models with only pre-operative score terms, circulation and depression (which chi-squared statistics are greater than 2000 in the models and coefficients are significantly large, i.e. greater than absolute value 200) were selected to be adjusted for the hip outcomes. Circulation, diabetes, and depression were selected for the knee outcomes based on the same criteria.

The 3rd degree left-hand-side-only model obtaining the maximum likelihood estimates is as below for the OHS:

5 $$ {y}_i^{\theta }={\beta}_0+{\beta}_1{x}_i+{\beta}_2{x}_i^2+{\beta}_3{x}_i^3+{\gamma}_1{z}_{1i}+{\gamma}_2{z}_{2i}+{\gamma}_3{z}_{3i}+{\gamma}_4{z}_{4i}+{\varepsilon}_i $$

where *ε*~*N*(0, *σ*^2^). *y* indicates the changed-operative score, and *x* indicates pre-operative score. *y* is subject to a Box-Cox transform with parameter *θ*. *z*_1_, *z*_2_, *z*_3_ are untransformed age, gender, circulation, and depression [26].

## Results

### Demographics

In total, 181,423 had hip replacement surgeries; over half (*N* = 106,493; 59%) were female with ages ranging from 13 to 100 years (SD 10.5; male, 15–99, SD 10.4), with a mean age of 68.6 years (male, 67.2 years). At baseline, of the total, 14% (*N* = 24,945) patients reported no comorbidity, 38.2% (*N* = 69,249) reported that they have one comorbidity, and 17.8% (*N* = 3234) have more than three comorbidities. 5.4% (*N* = 9866) reported circulation, diabetes 8.7% (*N* = 15,816), and depression 7.3% (*N* = 13,252).

For the knee replacement population, over half (*N* = 107,127; 56%) were female with ages ranging from 18 to 99 years (SD 9.1; male, 16–102, SD 8.6), with a mean age of 69.3 years (male, 69.3 years). At baseline, of the total, 9.3% (*N* = 17,712) patients reported no comorbidity, 33.3% (*N* = 63,804) reported that they have one comorbidity, and 23.6% (*N* = 45,200) have more than three comorbidities. Seven percent (*N* = 13,438) have reported circulation, diabetes 12.4% (*N* = 23,696), and depression 8.3% (*N* = 15,823) (Table 2).

**Table 2:** Baseline covariates

		Hip						Knee					
		Total	Patients' self-assessed transition level^a^					Total	Patients' self-assessed transition level				
			Much better	A little better	About the same	A little worse	Much worse		Much better	A little better	About the same	A little worse	Much worse
Age	*N*	181,423	155,899	15,565	3890	2382	1633	191,379	138,407	31,650	8985	7029	4610
	Mean (SD)	68.0 (10.5)	68.0 (10.4)	68.1 (11.2)	69.3 (10.9)	68.4 (10.8)	67.0 (11.1)	69.3 (8.9)	69.7 (8.7)	68.7 (9.2)	68.4 (9.5)	67.4 (9.2)	65.9 (9.5)
	Min, Max	(13, 100)	(13, 100)	(16, 98)	(16, 95)	(19, 97)	(16, 92)	(16, 102)	(16, 102)	(21, 100)	(26, 95)	(33, 95)	(28, 96)
Gender	Male	74,907 (41.3)	65,067 (41.7)	5927 (38.1)	1535 (39.5)	902 (37.9)	642 (39.3)	84,219 (44.0)	61,226 (44.2)	13,349 (42.2)	4007 (44.6)	3270 (46.5)	2118 (45.9)
	Female	106,493 (58.7)	90,810 (58.3)	9637 (61.9)	2356 (60.6)	1479 (62.1)	991 (60.7)	107,127 (56.0)	77,162 (55.8)	18,290 (57.8)	4977 (55.4)	3757 (53.5)	2492 (54.1)
Comorbidity	Heart disease	16,906 (9.3)	13,876 (8.9)	1765 (11.3)	517 (13.3)	292 (12.3)	218 (13.4)	20,100 (10.5)	13,583 (9.8)	3778 (11.9)	1135 (12.6)	920 (13.1)	610 (13.2)
	High blood pressure	71,267 (39.3)	60,621 (38.9)	6573 (42.2)	1656 (42.6)	973 (40.9)	650 (39.8)	88,549 (46.3)	63,700 (46.0)	15,181 (48.0)	4152 (46.2)	3177 (45.2)	2033 (44.1)
	Stroke	2432 (1.3)	1931 (1.2)	275 (1.8)	86 (2.2)	56 (2.4)	52 (3.2)	3130 (1.6)	2001 (1.5)	644 (2.0)	192 (2.1)	168 (2.4)	107 (2.3)
	Circulation	9866 (5.4)	7317 (4.7)	1532 (9.8)	429 (11.0)	259 (10.9)	187 (11.5)	13,438 (7.0)	7994 (5.8)	3184 (10.1)	950 (10.6)	756 (10.8)	494 (10.7)
	Lung disease	13,542 (7.5)	11,138 (7.1)	1443 (9.3)	361 (9.3)	253 (10.6)	203 (12.4)	16,165 (8.5)	11,118 (8.0)	2933 (9.3)	865 (9.6)	695 (9.9)	491 (10.7)
	Diabetes	15,816 (8.7)	12,939 (8.3)	1728 (11.1)	503 (12.9)	288 (12.1)	185 (11.3)	23,696 (12.4)	15,582 (11.3)	4683 (14.8)	1387 (15.4)	1190 (16.9)	765 (16.6)
	Kidney	3029 (1.7)	2502 (1.6)	338 (2.2)	77 (2.0)	41 (1.7)	26 (1.6)	3423 (1.8)	2352 (1.7)	635 (2.0)	180 (2.0)	150 (2.1)	90 (2.0)
	Nervous system	1342 (0.7)	1086 (0.7)	151 (1.0)	34 (0.9)	29 (1.2)	17 (1.0)	1845 (1.0)	1253 (0.9)	321 (1.0)	103 (1.2)	88 (1.3)	72 (1.6)
	Liver disease	929 (0.5)	768 (0.5)	94 (0.6)	31 (0.8)	13 (0.6)	14 (0.9)	997 (0.5)	636 (0.5)	206 (0.7)	64 (0.7)	53 (0.8)	33 (0.7)
	Cancer	8767 (4.8)	7431 (4.8)	811 (5.2)	212 (5.5)	115 (4.8)	90 (5.5)	9062 (4.7)	6571 (4.8)	1446 (4.6)	450 (5.0)	355 (5.1)	206 (4.5)
	Depression	13,252 (7.3)	10,247 (6.6)	1821 (11.7)	485 (12.5)	302 (12.7)	245 (15.0)	15,823 (8.3)	9693 (7.0)	3419 (10.8)	1073 (11.9)	894 (12.7)	680 (14.8)
	Arthritis	132,251 (72.9)	112,840 (72.4)	11,905 (76.5)	2953 (75.9)	1808 (75.9)	1243 (76.1)	151,394 (79.1)	108,910 (78.7)	25,624 (81.0)	7155 (79.6)	5508 (78.4)	3645 (79.1)
Comorbidity group	0	24,945 (13.8)	22,049 (14.1)	1724 (11.1)	451 (11.6)	277 (11.6)	179 (11.0)	17,712 (9.3)	13,345 (9.6)	2474 (7.8)	792 (8.8)	625 (8.9)	419 (9.1)
	1	69,249 (38.2)	61,012 (39.1)	5016 (32.2)	1197 (30.8)	752 (31.6)	500 (30.6)	63,804 (33.3)	47,914 (34.6)	9611 (30.4)	2599 (28.9)	2067 (29.4)	1356 (29.4)
	2	54,889 (30.3)	46,929 (30.1)	4957 (31.9)	1185 (30.5)	711 (29.9)	484 (29.6)	64,663 (33.8)	47,306 (34.2)	10,553 (33.3)	2973 (33.1)	2201 (31.3)	1421 (30.8)
	3+	32,341 (17.8)	25,909 (16.6)	3868 (24.9)	1058 (27.2)	642 (27.0)	470 (28.8)	45,200 (23.6)	29,842 (21.6)	9012 (28.5)	2621 (29.2)	2136 (30.4)	1414 (30.7)
Years of NHS PROMs	2009–2011	82,472 (45.5)	69,847 (44.8)	7498 (48.2)	1892 (48.6)	1120 (47.0)	801 (49.1)	87,809 (45.9)	62,121 (44.9)	15,316 (48.4)	4331 (48.2)	3378 (48.1)	2305 (50.0)
	2012–2015	98,952 (54.5)	86,052 (55.2)	8067 (51.8)	1999 (51.4)	1262 (53.0)	832 (51.0)	103,570 (54.1)	76,286 (55.1)	16,334 (51.6)	4654 (51.8)	3651 (51.9)	2305 (50.0)

^a^NHS PROMs post-operative question of success: Overall, how the problems now in the hip (knee) on which you had surgery, compared to before operation?

### Transition level

For the hip replacement surgery population, a great number of 155,899 (85.9%) patients answered *much better.* 15,565 (8.6%) patients answered *a little better.* Relatively smaller number of patients answered *about the same* 3891 (2.1%), *a little worse* 2382 (1.3%), and *much worse* 1633 (0.9%). For the knee replacement surgery population, 138,407 (72.3%) and 31,650 (16.5%) patients answered *much better* and *a little better,* respectively. 8985 (4.7%) patients answered *about the same*. 7029 (3.7%) patients answered *a little worse* and 4610 (2.4%) patients answered *much worse* (Table 3; Supplementary Table 1).

**Table 3:** The transition question (change score)

		Total	Much better	A little better	About the same	A little worse	Much worse
		*N* (%)	*N* (%)	*N* (%)	*N* (%)	*N* (%)	*N* (%)
Hip	OHS	179,370 (98.9)	155,899 (85.9)	15,565 (8.6)	3891 (2.1)	2382 (1.3)	1633 (0.9)
	EQ-5D-3L	169,549 (98.9)	147,638 (86.1)	14,577 (8.5)	3625 (2.1)	2216 (1.3)	1493 (0.9)
Knee	OKS	190,681 (100)	138,407 (72.3)	31,650 (16.5)	8985 (4.7)	7029 (3.7)	4610 (2.4)
	EQ-5D-3L	179,972 (100)	130,838 (72.5)	29,888 (16.6)	8434 (4.7)	6569 (3.6)	4243 (2.4)

Based on the question, Table 1

The Spearman’s rank correlation coefficients for the pre- and post-operative scores, *r*, are provided by the transition level in Table 4. The large correlations between of the pre- and post-operative scores are observed in patients with the transition level of *about the same*, *a little worse,* and *much worse.*

**Table 4:** Spearman’s rank correlation coefficients (95% CIs) for the change (pre- and post-operative) scores

		Total	Transition level^a^				
			Much better	A little better	About the same	A little worse	Much worse
Hip	*N*	181,424	155,899	15,565	3891	2382	1633
	OHS	0.32 (0.31, 0.32)	0.33 (0.32, 0.33)	0.55 (0.54, 0.56)	0.57 (0.55, 0.59)	0.57 (0.54, 0.60)	0.50 (0.46, 0.53)
	*N*	171,423	147,638	14,577	3625	2216	1493
	EQ-5D-3L	0.32 (0.31, 0.32)	0.31 (0.31, 0.32)	0.47 (0.46, 0.48)	0.50 (0.48, 0.53)	0.47 (0.43, 0.50)	0.45 (0.40, 0.49)
Knee	*N*	191,379	138,407	31,650	8985	7029	4610
	OKS	0.37 (0.37, 0.37)	0.36 (0.36, 0.37)	0.56 (0.55, 0.57)	0.61 (0.60, 0.63)	0.62 (0.61, 0.64)	0.57 (0.55, 0.58)
	*N*	180,546	130,838	29,888	8434	6569	4243
	EQ-5D-3L	0.37 (0.37, 0.37)	0.34 (0.34, 0.35)	0.48 (0.47, 0.49)	0.51 (0.49, 0.52)	0.49 (0.47, 0.51)	0.46 (0.44, 0.49)

^a^Based on the question, Table 1

### Univariate responsiveness measures for the paired data

The OHS and the OKS showed great univariate responsiveness in total, i.e. SRM [Eq. 1], SES [Eq. 2], and RI [Eq. 3] in total: 1.8, 2.8, and 0.6 (~ 0.7) in the OHS and 1.4, 2.5, and 0.7 in the OKS. In addition, the OHS and the OKS showed distinctive differences in the SRM [Eq. 1] by the 3-level transition, in particular, *a little better* vs. *about the same* vs. *much worse*: 1.5 (~ 1.6) vs. 0.8 (~ 0.9) vs. 0.3 (~ 0.4) in the OHS and 1.5 vs. 0.8 (~ 0.9) vs. 0.3 (~ 0.4) in the OKS. There was little difference among the 3-level transition for the SES: 1.7 vs. 1.3 (~ 1.4) vs. 1 (~ 1.1) in the OHS and 1.7 vs. 1.2 vs. 1 in the OKS (Tables 5 and 6)*.*

**Table 5:** Hip – the SRM, SES, and RI (with 95% CIs) for the OHS and the EQ-5D-3L (by the transition)

			Total	Much better	A little better	About the same	A little worse	Much worse
OHS [0, 48]	Change	*N*	181,424	155,899	15,565	3891	2382	1633
		(Post-op. mean)	39.3	41.3	28.8	24.2	21.6	14.3
		(Pre-op. mean)	18.2	18.3	17.3	17.8	18.9	16.7
		Mean (SD)	21.1 (9.9)	22.9 (8.6)	11.5 (7.8)	6.3 (8.0)	2.7 (7.6)	− 2.3 (8.3)
	Mean_change score_/SD_change score_		2.1 (2.1, 2.1)	2.7 (2.7, 2.7)	1.5 (1.5, 1.5)	0.8 (0.8, 0.8)	0.4 (0.3, 0.4)	− 0.3 (− 0.3, − 0.2)
	**SRM [Eq.** **1****]**		1.8 (1.8, 1.8)^a^	2.3 (2.3, 2.3)	1.6 (1.5, 1.6)	0.9 (0.8, 0.9)	0.4 (0.3, 0.4)	− 0.3 (− 0.3, − 0.2)
	**SES [Eq.** **2****]**		2.8 (2.8, 2.8)^b^	2.9 (2.9, 2.9)	1.7 (1.7, 1.7)	1.4 (1.3, 1.4)	1.0 (1.0, 1.1)	1.1 (1, 1.2)
	**RI [Eq.** **3****]**		0.6 (0.6, 0.7)^c^					
EQ-5D-3L [− 0.59, 1]	Change	*N*	171,423	147,638	14,577	3625	2216	1493
		(Post-op. mean)	0.8	0.8	0.6	0.5	0.4	0.2
		(Pre-op. mean)	0.4	0.4	0.3	0.3	0.4	0.3
		Mean (SD)	0.4 (0.3)	0.5 (0.3)	0.3 (0.3)	0.2 (0.3)	0.1 (0.3)	− 0.1 (0.3)
	Mean_change score_/SD_change score_		1.3 (1.3, 1.3)	1.4 (1.4, 1.5)	0.8 (0.8, 0.9)	0.5 (0.4, 0.5)	0.2 (0.1, 0.2)	− 0.3 (− 0.4, − 0.3)
	**SRM [Eq.** **1****]**		1.1 (1.1, 1.1)	1.2 (1.2, 1.2)	0.8 (0.8, 0.8)	0.5 (0.5, 0.5)	0.2 (0.1, 0.2)	− 0.3 (− 0.4, − 0.3)
	**SES [Eq.** **2****]**		1.6 (1.6, 1.6)	1.6 (1.6, 1.6)	1.4 (1.4, 1.4)	1.3 (1.3, 1.3)	1.1 (1.1, 1.1)	1.2 (1.1, 1.3)
	**RI [Eq.** **3****]**		0.3 (0.3, 0.4)					

^a^The SRM [Eq. 1] of the total OHS = $\frac{{\mathrm{Mean}}_{\mathrm{change}\ \mathrm{score}}/{\mathrm{SD}}_{\mathrm{change}\ \mathrm{score}}}{\surd 2\times \surd \left(1-r\right)}=\frac{2.1}{\surd 2\times \surd \left(1-0.32\right)}=1.8$

^b^The SES [Eq. 2] of the total OHS = $\frac{{\mathrm{Mean}}_{\mathrm{pre}-\mathrm{op}.\mathrm{score}}-{\mathrm{Mean}}_{\mathrm{post}-\mathrm{op}.\mathrm{score}\ \left(\mathrm{of}\ \mathrm{the}\ \mathrm{success}\ \mathrm{level}\right)}}{{\mathrm{SD}}_{\mathrm{pre}-\mathrm{op}.\mathrm{score}\ \left(\mathrm{of}\ \mathrm{the}\ \mathrm{success}\ \mathrm{level}\right)}}=\frac{18.2-40.46}{7.87}=\left|2.83\right|=2.8$

where 18.2 is pre-op. mean of the OHS (Table 5), 40.46 (7.42) is post-op. mean (SD), and 17.87 (7.87) is pre-op. of mean (SD) of the total improved subjects (*N* = 169,142); 40.46 of the post-op. mean and 7.87 of the pre-op. SD were used in this case

^c^The RI [Eq. 3] of the total OHS = $\frac{{\mathrm{MCID}}_{\mathrm{anchor}-\mathrm{based}}}{{\mathrm{SD}}_{\mathrm{change}\ \mathrm{score}\ \left(\mathrm{of}\ \mathrm{the}\ \mathrm{stable}\ \mathrm{level}\right)}}=\frac{{\mathrm{Mean}}_{\mathrm{change}\ \mathrm{score}}\ \mathrm{of}\ \mathrm{A}\ \mathrm{little}\ \mathrm{better}\ \mathrm{vs}.\mathrm{About}\ \mathrm{the}\ \mathrm{same}\ }{{\mathrm{SD}}_{\mathrm{change}\ \mathrm{score}\ \left(\mathrm{of}\ \mathrm{the}\ \mathrm{stable}\ \mathrm{level}\right)}}=\frac{5.15}{7.97}=0.6$

where the mean change of *a little better* vs. *about the same* is 5.15, and the mean (SD) of *about the same* is 6.34 (7.97)

**Table 6:** Knee – the SRM, SES, and RI (with 95% CIs) for the OKS and the EQ-5D-3L (by the transition)

			Total	Much better	A little better	About the same	A little worse	Much worse
OKS [0, 48]	Change	*N*	191,379	138,407	31,650	8985	7029	4610
		(Post-op. mean)	34.9	38.8	28.0	22.7	20.2	13.7
		(Pre-op. mean)	19.1	19.6	18.1	17.7	18.3	16.7
		Mean (SD)	15.8 (9.8)	19.2 (8.0)	9.9 (7.1)	5 (6.8)	1.9 (6.7)	− 3.0 (7.0)
	Mean_change score_/SD_change score_		1.6 (1.6, 1.6)	2.4 (2.4, 2.4)	1.4 (1.4, 1.4)	0.7 (0.7, 0.8)	0.3 (0.3, 0.3)	− 0.4 (− 0.5, − 0.4)
	**SRM [Eq.** **1****]**		1.4 (1.4, 1.4)	2.1 (2.1, 2.1)	1.5 (1.5, 1.5)	0.8 (0.8, 0.9)	0.3 (0.3, 0.4)	− 0.5 (− 0.5, − 0.4)
	**SES [Eq.** **2****]**		2.5 (2.5, 2.5)	2.7 (2.7, 2.7)	1.7 (1.7, 1.7)	1.2 (1.2, 1.2)	1.0 (1.0, 1.0)	0.9 (0.8, 0.9)
	**RI [Eq.** **3****]**		0.7 (0.7, 0.7)					
EQ-5D-3L [− 0.59, 1]	Change	*N*	180,546	130,838	29,888	8434	6569	4243
		(Post-op. mean)	0.7	0.8	0.6	0.5	0.4	0.2
		(Pre-op. mean)	0.4	0.4	0.4	0.4	0.4	0.3
		Mean (SD)	0.3 (0.3)	0.4 (0.3)	0.2 (0.3)	0.1 (0.3)	0.1 (0.3)	− 0.1 (0.4)
	Mean_change score_/SD_change score_		1.0 (0.9, 1.0)	1.2 (1.2, 1.2)	0.8 (0.8, 0.8)	0.4 (0.4, 0.5)	0.2 (0.1, 0.2)	− 0.4 (− 0.4, − 0.4)
	**SRM [Eq.** **1****]**		0.8 (0.8, 0.9)	1.0 (1.0, 1.1)	0.8 (0.7, 0.8)	0.5 (0.4, 0.5)	0.2 (0.1, 0.2)	− 0.4 (− 0.4, − 0.4)
	**SES [Eq.** **2****]**		1.3 (1.3, 1.3)	1.4 (1.4, 1.4)	1.2 (1.2, 1.2)	1.3 (1.3, 1.3)	1.2 (1.2, 1.2)	1.2 (1.2, 1.3)
	**RI [Eq.** **3****]**		0.3 (0.3, 0.3)					

The univariate responsiveness in total for the generic instrument EQ-5D-3L were 1.1, 1.6, and 0.3 (~ 0.4) for the hip and 0.8 (~ 0.9), 1.3, and 0.3 for the knee replacement. The SRMs [Eq. 1] by the 3-level transition were 0.8 vs. 0.5 vs. 0.1 (~ 0.2) for the hip and 0.7 (~ 0.8) vs. 0.4 (~ 0.5) vs. 0.1 (~ 0.2) for the knee replacement. The SES values were similar to each other among the 3-level transition: 1.4 vs. 1.3 vs. 1.1 for the hip and 1.2 vs. 1.3 vs. 1.2 for the knee replacement.

The RI [Eq. 3] was calculated in total only as the calculation incorporates with the 2-level transition (i.e. *a little better* vs. *about the same)* in it. The RIs [Eq. 3] in total were 0.6 (~ 0.7) in the OHS and 0.7 in the OKS, which are moderate practical effects by Cohen’s thresholds (i.e. > 0.8 large, 0.5 to 0.8 moderate, and < 0.5 small) [21, 27]. The RIs [Eq. 3] in total for EQ-5D-3L showed negligible practical effects, 0.3 (~ 0.4) for the hip and 0.3 for the knee replacement. The SRM [Eq. 1] and SES [Eq. 2] can be interpreted similarly. The SRM [Eq. 1] and SES [Eq. 2] of ‘A little better’ in the OHS were 1.6 and 1.7, respectively. Both can be interpreted as a crucial difference in the ‘successful’ percentage in each of the two groups (*r*) of 0.62 [28]. The SRM [Eq. 1] and SES [Eq. 2] of ‘A little better’ in the EQ-5D-3L were 0.8 and 1.4, respectively, which can be interpreted as moderate and crucial differences in the ‘successful’ percentage in each of the two groups (*r*) of 0.37 and 0.57 [28]. This implies the SRM [Eq. 1] shows a good discriminative ability for the different severities in comparison to the SES [Eq. 2], and EQ-5D-3L is less responsive in comparison to the OHS.

### The paired data-specific MCID as the threshold for improvement

The paired data-specific MCID [Eq. 4] was calculated, applying the SRM [Eq. 1] as a desired ES. Multivariate responsiveness was examined using the defined capacity of benefit score as improvement (i.e. 22 for the OHS, and 0.428 for the hip EQ-5D-3L; 16 for the OKS and 0.309 for the knee EQ-5D-3L)^2^, adjusting covariates. Various ways to assess the improvement for the independent data are presented in Supplementary Table 2. Those scores are smaller than the capacity of benefit scores for the paired data. The SRM applied MCIDs for the independent data are 6 for the OHS, and 0.196 for the hip EQ-5D-3L, using Cohen’s medium (0.5) effect size. The MDCs (minimal detectable changes, defined as the minimal change that falls beyond the measurement error in the measurement score [29]) are 6 for the OHS and 0.234 for the hip EQ-5D-3L, with ICC 0.9. The anchor-based MCIDs are 9 for the OHS, and 0.101 for the hip EQ-5D-3L, using the short distance. The mean change scores using the anchor are 6 for the OHS, and 0.106 for the hip EQ-5D-3L. A greater capacity of benefit score is required for the paired data in comparison to the independent data, to detect how likely the surgery is to distinguish an actual effect from one of chance in the pre- and post-operative outcomes.

### Multivariate responsiveness measures – observed and predicted improvement

The percentage improvements based on patients’ perceptions were high in the OHS and the OKS (Tables 7 and 8). The percentages of the observed (predicted) total improvement were 51 (54)% in the OHS and 73 (58)% in the OKS. In addition, the OHS and the OKS showed distinctive percentage differences by the 3-level transition, i.e. *a little better* vs. *about the same* vs. *a little worse.* As an example, the observed percentages of the 3-level transition were 10% vs. 4% vs. 1% in the OHS and 21% vs. 6% vs. 3% in the OKS. The percentages of the observed (predicted) total improvement in the generic instrument EQ-5D-3L were 44 (48)% for the hip and 42 (44)% for the knee replacement population. The observed (predicted) percentages of the 3-level transition in the EQ-5D-3L were 39 (41)% vs. 29 (11)% vs. 21 (4)% for the hip and 39 (45)% vs. 32 (36)% vs. 26 (14)% for the knee replacement population.

**Table 7:** Hip – patients’ perception of improvement (%) (using the paired data-specific MCID [Eq. 4])

Instrument		Measure	Total	Much better	A little better	About the same	A little worse	Worse
OHS			(*N* = 181,424)	(*N* = 155,899)	(*N* = 15,565)	(*N* = 3891)	(*N* = 2382)	(*N* = 1633)
	Observed		51%	58%	**10**%	**4**%	**1**%	1%
		AUC^a^	*0.8*	*0.8*	*0.8*	*0.8*	*0.7*	*0.8*
	Predicted	Pre-op.	53%	62%	-	-	-	-
		Pre-op., age, gender, comorbidity	54%	62%	-	-	-	-
EQ-5D-3L			(*N* = 171,423)	(*N* = 147,638)	(*N* = 14,577)	(*N* = 3625)	(*N* = 2216)	(*N* = 1493)
	Observed		44%	46%	**39**%	**29**%	**21**%	13%
		AUC	*0.9*	*0.9*	*0.9*	*0.8*	*0.8*	*0.7*
	Predicted	Pre-op.	48%	47%	43%	11%	4%	6%
		Pre-op., age, gender, comorbidity	48%	47%	41%	11%	4%	6%

^a^Area under the receiver operating characteristic (ROC) curve

**Table 8:** Knee – patients’ perception of improvement (%) (using the paired data-specific MCID [Eq. 4])

Instrument		Measure	Total	Much Better	A little better	About the same	A little worse	Worse
OKS			(*N* = 191,379)	(*N* = 138,407)	(*N* = 31,650)	(*N* = 8985)	(*N* = 7029)	(*N* = 4610)
	Observed		73%	68%	**21**%	**6**%	**3**%	1%
		AUC	*0.7*	*0.8*	*0.7*	*0.7*	*0.7*	*0.7*
	Predicted	Pre-op.	58%	74%	-	-	-	-
		Pre-op., age, gender, comorbidity	58%	75%	-	-	-	-
EQ-5D-3L			(*N* = 180,546)	(*N* = 130,838)	(*N* = 29,888)	(*N* = 8434)	(*N* = 6569)	(*N* = 4243)
	Observed		42%	45%	**39**%	**32**%	**26**%	14%
		AUC	*0.9*	*0.9*	*0.9*	*0.8*	*0.8*	*0.7*
	Predicted	Pre-op.	44%	46%	46%	35%	8%	5%
		Pre-op., age, gender, comorbidity	44%	44%	45%	36%	14%	5%

The observed (predicted) percentage improvements applied the Cohen’s ES (0.5 and 0.8) are additionally provided in Supplementary Table 3 and 4 for the independent data. The observed (predicted) percentages for the medium improvement were 93 (99)% in the OHS, and 85 (98)% in the OKS. The observed (predicted) percentage improvements in the EQ-5D-3L were 75 (74)% for the hip and 60 (58)% for the knee replacement population. The observed (predicted) percentages of the 3-level transition were 78 (90)% vs. 52 (57)% vs. 34 (19)% in the OHS, and 73 (85)% vs. 46 (42)% vs. 29 (8)% in the OKS. The observed (predicted) percentages of the 3-level transition in the EQ-5D-3L were 50 (52)% vs. 38 (50)% vs. 29 (41)% for the hip and 45 (48)% vs. 36 (47)% vs. 29 (42)% for the knee replacement population.

A great number of patients (86% for hip and 72% for knee) answered *much better* for success of the surgery (Table 2). In addition, the greater capacity of benefit score was applied for the calculation of the paired data-specific percentage improvement. Therefore, overall percentages (%) of patients’ perception of improvement are lower in comparison to the improvement for the independent data*.* There were much distinctive percentage differences by the transition level when the paired data-specific capacity of benefit score was applied for the calculation.

### Model performance

The area under the ROC curve (AUC) with 95% binomial exact confidence intervals was calculated to examine discriminative ability with each MCID assuming as the true improvement status, using the patient rating instruments, i.e. OHS, OKS, and EQ-5D-3L (Tables 7 and 8) for the observational data.

### Internal validation

Internal validation was performed by examining what sensitivity there is within the dataset to the period: NHS PROMs linked to HES 2009–2011 vs. 2012–2015. There was no significant sensitivity by two-period (Supplementary Figure 2).

## Discussion

The paired data-specific sensitivity of the EQ-5D-3L, the OHS and the OKS were investigated to detect changes in the health state over time for the population who underwent hip or knee surgeries in the UK. To ensure accuracy of the health status and instrument evaluation in hip and/or knee replacement surgery, the paired data-specific SRM was examined for the univariate responsiveness. In addition, the SES and the RI were calculated using the patients' self-assessed transition. Multiple responsiveness metrics were applied, including a robust modelling approach that adjusted significant baseline covariates to estimate percentage improvements. From the modelling approach, the paired data-specific observed (and the predicted) percentages of improvement were distinctive by the transition level (Tables 7 and 8). The multivariate modelling method provided robust responsiveness statistics in terms of adjusting the patient demographic information and comorbidities. Responsiveness from the models was interpretable with a percentage scale of improvement.

A greater capacity of benefit score is applied to a calculation of improvement for a paired data. Therefore, overall percentages (%) of patients’ perception of improvement are relatively low. The missing cases of predicted improvement by certain transition levels are inevitable for the Oxford questionnaires which have ceiling effects where a greater study population answered *much better* after the surgery.

Disease-specific and generic instruments are both available in the PROMS data in the UK, and they showed reasonable responsiveness as a health-related instrument that measures functional state. A previous study using the NHS patient-reported outcome measures (PROMs) supports moderate correlations (0.3 to 0.6) between the EQ-5D-3L and other measures of patient-reported health changes, including the OHS and the OKS [30]. Nonetheless, there has been a lack of evidence to support the ability to discriminate. In terms of detecting clinically significant changes in arthroplasty surgery, although it has not been firmly fixed yet, a number of studies indicated that disease-specific instruments are more responsive than generic instruments [4, 31–35]. The present study showed that, although the responsiveness was greater and more distinctive in the disease-specific instruments, the responsiveness of the EQ-5D-3L for hip and knee surgery are reasonably good. The EQ-5D would be useful in terms of short completion time and good validity [3]. Nevertheless, it may not be sufficiently sensitive to be used solely in hip and/or knee replacement surgery, either to discriminate between cases of differing severities by a transition question or to detect the changes in severity or functional status over time [21].

The accurate identification and the early stage of stratification of patients undergoing hip and/or knee replacement are one of the greatest unmet needs. A robust and precise measurement instrument will be effective in the management of arthroplasty surgery for particular group of patients. The OHS and the OKS have been provided evidence that the instruments are able to contribute to the better management of arthroplasty surgery. In general, arthroplasty surgery is based on an individual level in terms of a patient’s expectations, symptoms, diagnoses, and degree of pain. Although the excellence of the Oxford questionnaires over other patient-reported questionnaires was examined, the Oxford questionnaires have a ceiling effect, and the threshold levels are always a trade-off between sensitivity and specificity. Moreover, the current version of the OHS or the OKS does not contain a psychological measurement such as depression or anxiety which is also important in health outcome. Further investigation is required about their potential roles of clinical or trial use, cost-effectiveness, and their effects on referral patterns.

### Strengths and limitations

The strength of this study includes using a large cohort data linked to HES on both hip and knee replacement surgeries that provided enough power to support the research outcomes. Although the sample size is large enough to validate the improvement values using complete-case analysis, validation by an external data set was not conducted. The study design may be suboptimal compared to a well-blinded randomized clinical trial. Additional care may be required in the interpretation of patients’ socio-demographics, clinical/treatment and other unobserved covariates that may not be adjusted.

A secondary transition was not used in the study. The NHS PROMs data contains only one-point transition measurement (6 months post-operation) and a more objective point assessment may need to be considered [36]. The mean change score using a patient-reported transition (i.e. an anchor approach) has a limitation, in that the one-point transition measurement relies on a patient’s memory in global health status, and it could be a more subjective change measurement in contrast with each of the pre- and post- point assessments [36]. In addition, the measurement errors should account for repeatedly measured patient-reported outcomes. There will be several ways to control the errors such as use of the MDC approach (i.e. the threshold for improvement adjusted for measurement error) or applying advance statistical inference approaches such as Bayesian models with computational methods. Potential limitations or difficulties would be the fact that it is not easy to precisely estimate a percentage improvement using the model fitting with the EQ-5D-3L due to the nature of the real number scales (− 0.59 to 1), and the scale is very dispersed (Supplementary Figure 3).

## Conclusions

The paired data-specific responsiveness was investigated in the population from the NHS PROMs data who underwent hip or knee surgery in the UK. The OHS and the OKS showed good discriminative abilities in the clinically significant changes, and the EQ-5D-3L also showed comparatively moderate responsiveness. Using the paired data-specific capacity of benefit scores, the OHS and the OKS showed distinctive differences of clinically significant chances by the level of the transition, in particular for the 3-level transition, i.e. *a little better, about the same,* and *a little worse*. This is useful in clinical practice as rationale for access to surgery at the individual-patient level. The study finding supports the idea of using a precise estimation of improvement and appropriate instruments in arthroplasty surgery. It seems that a generic measure would be beneficial to use along with the disease-specific instruments in terms of cross-validation unless an enhanced instrument has been developed, or a specific reason is required in the reporting system.

## Supplementary Information

**Additional file 1: Supplementary Table 1.** Descriptive statistics. **Supplementary Table 2.** The estimated improvement by other definitions for the independent data. **Supplementary Table 3.** Hip – patients’ perception of improvement (%) (using the Cohen’s ES (0.5 and 0.8) applied MCID). **Supplementary Table 4.** Knee – patients’ perception of improvement (%) (using the Cohen’s ES (0.5 and 0.8) applied MCID). **Supplementary Figure 1.** The OKS and EQ-5D-3L – total population (1, 3) and the transition level (2, 4). **Supplementary Figure 2.** The hip (1, 2) and the knee (3, 4) – total population in the NHS PROMs years. **Supplementary Figure 3.** Histograms of the OHS and the OKS changes (1, 3); Histograms of the EQ-5D-3L changes showed multimodal distributions (2, 4). **Supplementary Figure 4.** The OHS (1, 3, 5, 7, 9) and the OKS (2, 4, 6, 8, 10) proportion and probabilities of improvement by the transition level in the 4th degree fractional polynomial logistic regressions (using the Cohen’s medium ES (0.5) applied MCID).

## Data Availability

The datasets generated and/or analysed during the current study are not publicly available due to the licence. The online supplementary document for research findings is available.

## Abbreviations

OHS: Oxford Hip Score
OKS: Oxford Knee Score
PROMs: Patient-reported outcome measures
HES: Hospital Episodes Statistics
SRM: Standardized response mean
SES: Standardized effect size
RI: Responsiveness index
MCID: Minimally clinically important difference
MDC: Minimal detectable change
ROC: Receiver operating characteristic
AUC: Area under the ROC curve

## Footnotes

1 A couple of patients (*N* = 230) that have the same procedure (as identified by “PROMS_PROC_CODE”) carried out on the same side of the body more than once, e.g. two primary hip replacements of the left hip. It was decided that it might be best to treat them as revisions; these duplicates were further sorted by the variables “EPISODE_MATCH_RANK” and “EPIKEY”. The former variable will ensure that the highest quality match between HES and PROMs is listed first, and the latter variable will ensure that a unique sorting is produced. Based on this sorting, the second observations were dropped out from the duplicates one (*N* = 602,287).

2 Rounded up to nearest whole number: 21.1 for the OHS and 15.8 for the OKS
