# Correction to: Assessing responsiveness of the EQ-5D-3L, the Oxford Hip Score, and the Oxford Knee Score in the NHS patient-reported outcome measures

**DOI:** 10.1186/s13018-021-02273-0

**Published:** 2021-02-05

**Authors:** Sujin Kang

**Affiliations:** Faculty of Medicine, Imperial College London, South Kensington Campus, London, SW7 2AZ UK

**Correction to: J Orthop Surg Res (2021) 16:18**

**https://doi.org/10.1186/s13018-020-02126-2**

Following publication of the original article [1], the author notified us that the Supplementary document should be updated.

The original article has been corrected.
