# Supplementary material for: Assessing responsiveness of the EQ-5D-3L, the Oxford Hip Score, and the Oxford Knee Score in the NHS patient-reported outcome measures
Source: J Orthop Surg Res. 2021 Jan 7;16:18. doi: 10.1186/s13018-020-02126-2 (PMC7791983; doi:10.1186/s13018-020-02126-2)
Supplement: Supplementary file 1 — Additional file 1: Supplementary Table 1. Descriptive statistics. Supplementary Table 2. The estimated improvement by other definitions for the independent data. Supplementary Table 3. Hip – patients’ perception of improvement (%) (using the Cohen’s ES (0.5 and 0.8) applied MCID). Supplementary Table 4. Knee – patients’ perception of improvement (%) (using the Cohen’s ES (0.5 and 0.8) applied MCID). Supplementary Figure 1. The OKS and EQ-5D-3L – total population (1, 3) and the transition level (2, 4). Supplementary Figure 2. The hip (1, 2) and the knee (3, 4) – total population in the NHS PROMs years. Supplementary Figure 3. Histograms of the OHS and the OKS changes (1, 3); Histograms of the EQ-5D-3L changes showed multimodal distributions (2, 4). Supplementary Figure 4. The OHS (1, 3, 5, 7, 9) and the OKS (2, 4, 6, 8, 10) proportion and probabilities of improvement by the transition level in the 4th degree fractional polynomial logistic regressions (using the Cohen’s medium ES (0.5) applied MCID). [file 13018_2020_2126_MOESM1_ESM.docx]

**Supplementary document**

Supplementary Table 1. Descriptive statistics

|  |  | Variable |  | N | Mean (SD) | Min | Max |
| --- | --- | --- | --- | --- | --- | --- | --- |
| Hip | OHS | Pre-op. |  | 181,424 | 18.2 (8.1) | 0 | 48 |
|  |  | Post-op. |  | 181,424 | 39.3 (8.8) | 0 | 48 |
|  |  | Change score | Transition question | 181,424 | 21.1 (9.9) | -35 | 48 |
|  |  |  | *Much better* | 155,899 | 22.9 (8.6) | -28 | 48 |
|  |  |  | *A little better* | 15,565 | 11.5 (7.8) | -26 | 44 |
|  |  |  | *About the same* | 3,891 | 6.3 (8.0) | -25 | 42 |
|  |  |  | *A little worse* | 2,382 | 2.7 (7.6) | -29 | 35 |
|  |  |  | *Much worse* | 1,633 | 2.3 (8.3) | -35 | 35 |
|  | EQ-5D-3L Index | Pre-op. |  | 176,455 | 0.4 (0.3) | -0.594 | 1 |
|  |  | Post-op. |  | 176,068 | 0.8 (0.2) | -0.594 | 1 |
|  |  | Change score | Transition question | 171,423 | 0.4 (0.3) | -1.285 | 1.594 |
|  |  |  | *Much better* | 147,638 | 0.5 (0.3) | -1.110 | 1.594 |
|  |  |  | *A little better* | 14,577 | 0.3 (0.3) | -0.873 | 1.285 |
|  |  |  | *About the same* | 3,625 | 0.2 (0.3) | -1.110 | 1.163 |
|  |  |  | *A little worse* | 2,216 | 0.1 (0.3) | -1.040 | 1.051 |
|  |  |  | *Much worse* | 1,493 | -0.1 (0.3) | -1.285 | 1.016 |
| Knee | OKS | Pre-op. |  | 191,379 | 19.1 (7.7) | 0 | 48 |
|  |  | Post-op. |  | 191,379 | 34.9 (9.7) | 0 | 48 |
|  |  | Change score | Transition question | 191,379 | 15.8 (9.8) | -30 | 47 |
|  |  |  | *Much better* | 138,407 | 19.2 (8.0) | -24 | 47 |
|  |  |  | *A little better* | 31,650 | 9.9 (7.1) | -30 | 41 |
|  |  |  | *About the same* | 8,985 | 5.0 (6.8) | -27 | 33 |
|  |  |  | *A little worse* | 7,029 | 1.9 (6.7) | -26 | 27 |
|  |  |  | *Much worse* | 4,610 | -3.0 (7.0) | -29 | 39 |
|  | EQ-5D-3L Index | Pre-op. |  | 186,093 | 0.4 (0.3) | -0.594 | 1 |
|  |  | Post-op. |  | 185,433 | 0.7 (0.3) | -0.594 | 1 |
|  |  | Change score | Transition question | 180,546 | 0.3 (0.3) | -1.285 | 1.429 |
|  |  |  | *Much better* | 130,838 | 0.4 (0.3) | -0.945 | 1.429 |
|  |  |  | *A little better* | 29,888 | 0.2 (0.3) | -1.146 | 1.250 |
|  |  |  | *About the same* | 8,434 | 0.1 (0.3) | -1.181 | 1.214 |
|  |  |  | *A little worse* | 6,569 | 0.1 (0.3) | -1.104 | 1.110 |
|  |  |  | *Much worse* | 4,243 | -0.1 (0.4) | -1.285 | 1.016 |

Supplementary Table 2. The estimated improvement by other definitions for the independent data

|  |  | MCID [D] | | MDC  (at 90% confidence level) | | | Anchor-based^4^ MCID | | Mean change score using the anchor |
| --- | --- | --- | --- | --- | --- | --- | --- | --- | --- |
| Hip | OHS | SRM MCID | 2.1 | ICC^3^ | 0.9 | 6 | Youden Index | 8 | 6 |
|  |  | M MCID^1^ | 6 |  | 0.8 | 9 | Short distance^5^ | 9 |  |
|  |  | L MCID^2^ | 10 |  | 0.7 | 11 |  |  |  |
|  | EQ-5D-3L | SRM MCID | 1.280 | ICC | 0.9 | 0.234 | Youden Index | 0.025/0.033/0.036 | 0.106 |
|  |  | M MCID | 0.196 |  | 0.8 | 0.331 | Short distance | 0.101 |  |
|  |  | L MCID | 0.313 |  | 0.7 | 0.405 |  |  |  |
| Knee | OKS | SRM MCID | 1.6 | ICC | 0.9 | 6 | Youden Index | 7 | 6 |
|  |  | M MCID | 6 |  | 0.8 | 8 | Short distance | 8 |  |
|  |  | L MCID | 9 |  | 0.7 | 10 |  |  |  |
|  | EQ-5D-3L | SRM MCID | 0.952 | ICC | 0.9 | 0.227 | Youden Index | 0.036 | 0.090 |
|  |  | M MCID | 0.182 |  | 0.8 | 0.321 | Short distance | 0.069 |  |
|  |  | L MCID | 0.292 |  | 0.7 | 0.393 |  |  |  |

^1^ MCID using Cohen’s medium (0.5) ES for the independent data
^2^ MCID using Cohen’s large (0.8) ES for the independent data

^3^ ICC (test-retest score reliabilities): Assumed previously reported over 0.9 for OHS/OKS using PROMs (2009-11) (Beard, et al., 2015)
^4^ Anchor-based: Transition question - *A little better* vs. *About the same*

^5^ Short distance: ($\sqrt{{(1-sensitivity)}^{2}{+(1-specificity)}^{2}}$)

The SRM applied MCIDs for independent data are 2.1 (OHS) and 1.28 (EQ-5D-3L) for hip, and 1.6 (OKS) and 0.95 (EQ-5D-3L) for knee (Supplementary Table 2). The MDC and the mean change score using the anchor are also displayed. The MCIDs for the independent data were calculated in using the Cohen’s ES (0.5 and 0.8) [E].
It displays improvement scores for the independent data by different definitions. The ranges of the improvement scores are 6 to 10 for the OHS and the OKS (ICC over 0.8, except SRM MCID); 0.2 to 0.33 for the hip and 0.2 to -0.32 for the knee population in EQ-5D-3L (ICC over 0.8, except SRM MCID).

***Multivariate responsiveness measures - Observed and predicted improvement***

Supplementary Table 3. Hip – Patients’ perception of improvement (%) (using the Cohen’s ES (0.5 and 0.8) for the independent data)

| Instrument | | Measure | Total | | *Much better* | | *A little better* | | *About the same* | | *A little worse* | | *Worse* | |  |
| --- | --- | --- | --- | --- | --- | --- | --- | --- | --- | --- | --- | --- | --- | --- | --- |
|  |  |  | M MCID | L MCID | M MCID | L MCID | M MCID | L MCID | M MCID | L MCID | M MCID | L MCID | M MCID | L MCID | |
| OHS |  |  | (N=181,424) | | (N=155,899) | | (N=15,565) | | (N=3,891) | | (N=2,382) | | (N=1,633) | |  |
|  | Observed |  | 93% | 88% | 98% | 94% | 78% | 60% | 52% | 33% | 34% | 17% | 14% | 6% | |
|  |  | *AUC* | *0.7* | *0.7* | *0.8* | *0.8* | *0.7* | *0.7* | *0.7* | *0.7* | *0.7* | *0.7* | *0.7* | *0.7* | |
|  | Predicted | Pre-op. | 99% | 96% | 100% | 97% | 90% | 70% | 60% | 11% | 17% | - | - | - | |
|  |  | Pre-op., age, gender, comorbidity | 99% | 96% | 99% | 97% | 90% | 69% | 57% | 15% | 19% | - | - | - | |
| EQ-5D-3L |  |  | (N=171,423) | | (N=147,638) | | (N=14,577) | | (N=3,625) | | (N=2,216) | | (N=1,493) | |  |
|  | Observed |  | 75% | 55% | 79% | 58% | 50% | 43% | 38% | 33% | 29% | 26% | 18% | 15% | |
|  |  | *AUC* | *0.7* | *0.9* | *0.7* | *0.9* | *0.9* | *0.9* | *0.8* | *0.8* | *0.8* | *0.8* | *0.7* | *0.7* | |
|  | Predicted | Pre-op. | 75% | 55% | 93% | 55% | 53% | 51% | 51% | 41% | 44% | 9% | 6% | 6% | |
|  |  | Pre-op., age, gender, comorbidity | 74% | 54% | 87% | 57% | 52% | 51% | 50% | 37% | 41% | 16% | 7% | 6% | |

Supplementary Table 4. Knee – Patients’ perception of improvement (%) (using the Cohen’s ES (0.5 and 0.8) for the independent data)

| Instrument | | Measure | Total | | *Much better* | | *A little better* | | *About the same* | | *A little worse* | | *Worse* | |  |
| --- | --- | --- | --- | --- | --- | --- | --- | --- | --- | --- | --- | --- | --- | --- | --- |
|  |  |  | M MCID | L MCID | M MCID | L MCID | M MCID | L MCID | M MCID | L MCID | M MCID | L MCID | M MCID | L MCID | |
| OKS |  |  | (N=191,379) | | (N=138,407) | | (N=31,650) | | (N=8,985) | | (N=7,029) | | (N=4,610) | |  |
|  | Observed |  | 85% | 78% | 96% | 91% | 73% | 58% | 46% | 30% | 29% | 16% | 9% | 4% | |
|  |  | *AUC* | *0.6* | *0.6* | *0.8* | *0.8* | *0.7* | *0.7* | *0.7* | *0.7* | *0.7* | *0.7* | *0.7* | *0.7* | |
|  | Predicted | Pre-op. | 98% | 94% | 99% | 96% | 85% | 63% | 42% | 2% | 5% | - | - | - | |
|  |  | Pre-op., age, gender, comorbidity | 98% | 93% | 99% | 96% | 85% | 65% | 42% | 5% | 8% | - | - | - | |
| EQ-5D-3L |  |  | (N=180,546) | | (N=130,838) | | (N=29,888) | | (N=8,434) | | (N=6,569) | | (N=4,243) | |  |
|  | Observed |  | 60% | 52% | 69% | 58% | 45% | 41% | 36% | 32% | 29% | 26% | 17% | 15% | |
|  |  | *AUC* | *0.7* | *0.8* | *0.7* | *0.8* | *0.9* | *0.9* | *0.8* | *0.8* | *0.8* | *0.8* | *0.7* | *0.7* | |
|  | Predicted | Pre-op. | 63% | 44% | 61% | 46% | 47% | 46% | 48% | 43% | 45% | 12% | 5% | 5% | |
|  |  | Pre-op., age, gender, comorbidity | 58% | 45% | 67% | 45% | 48% | 46% | 47% | 40% | 42% | 19% | 6% | 5% | |

***Model performance***

1 2


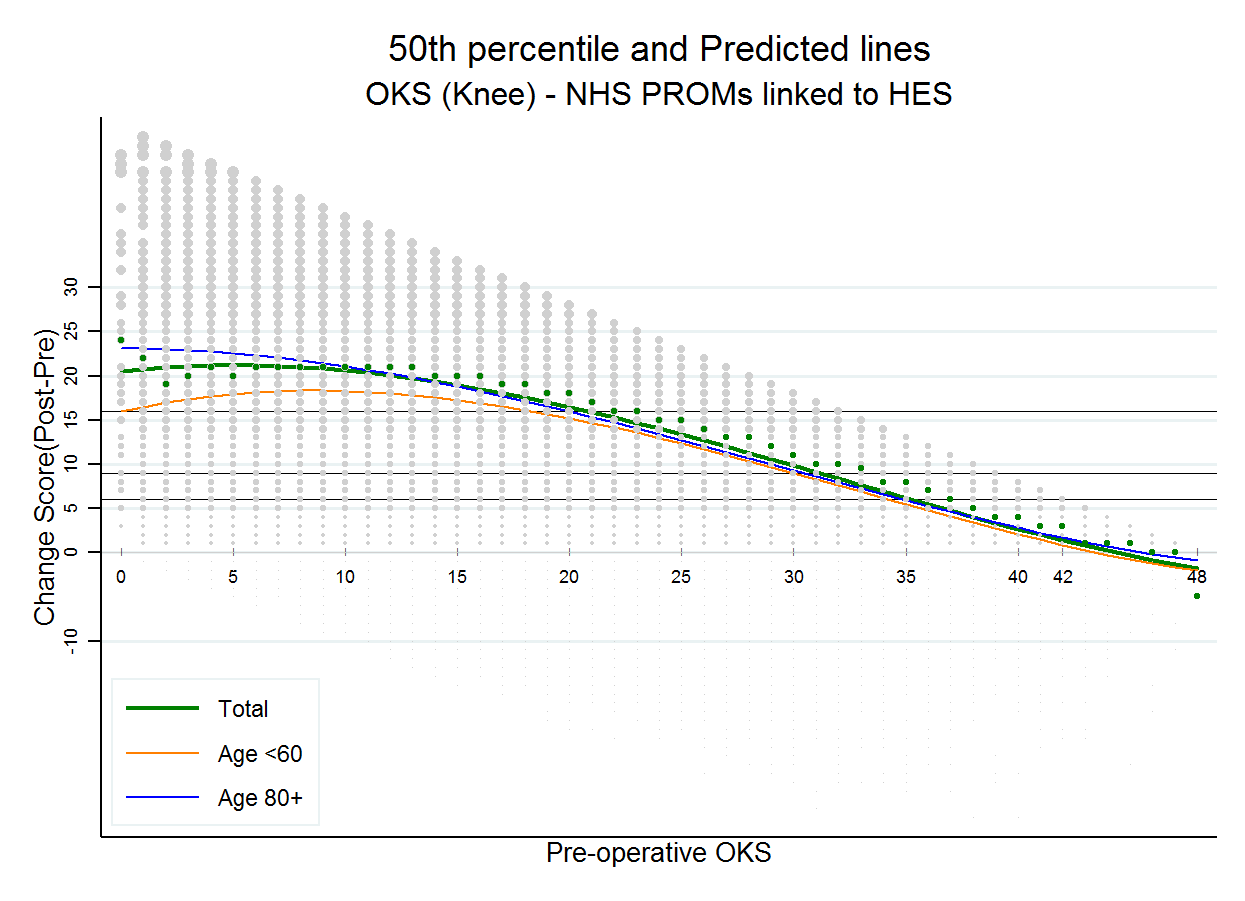

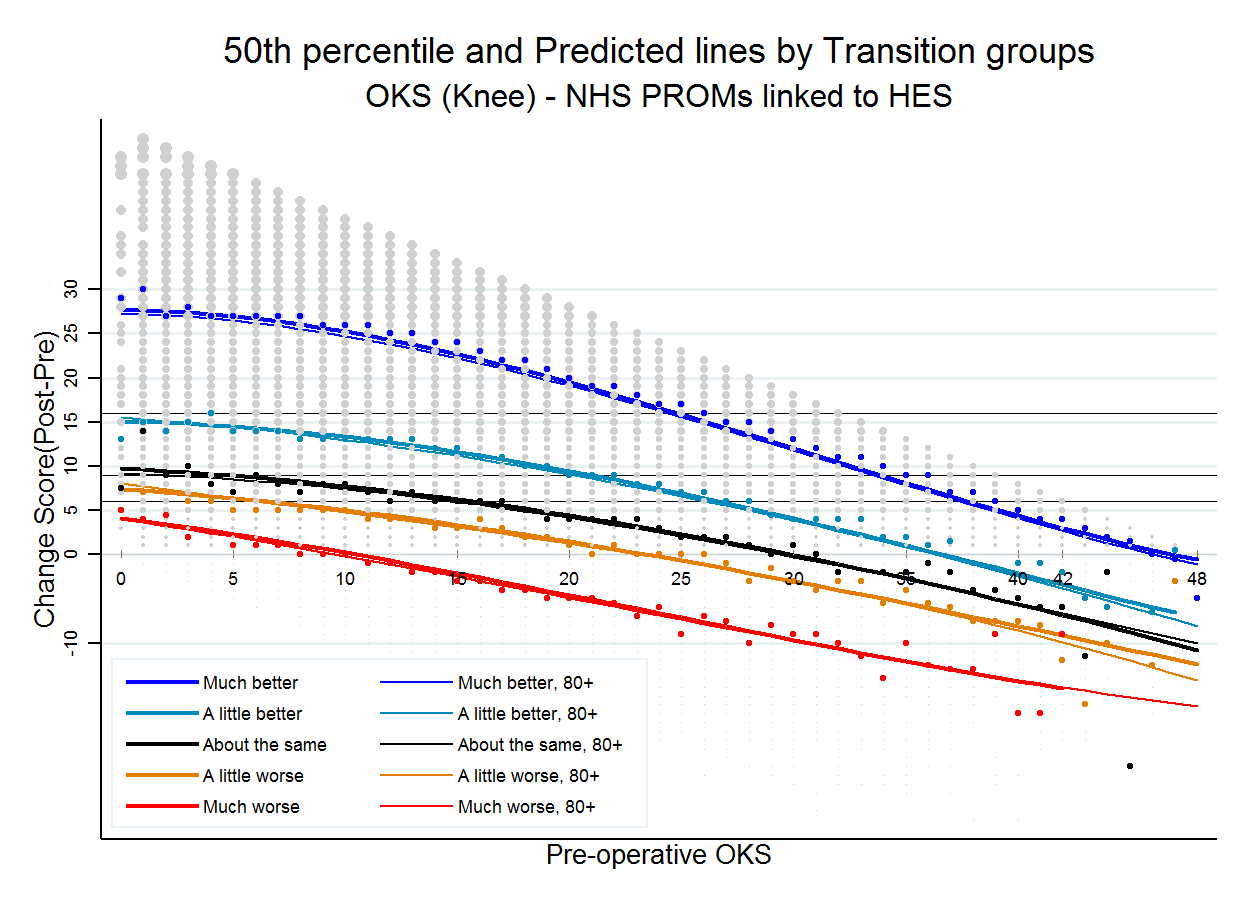


3 4


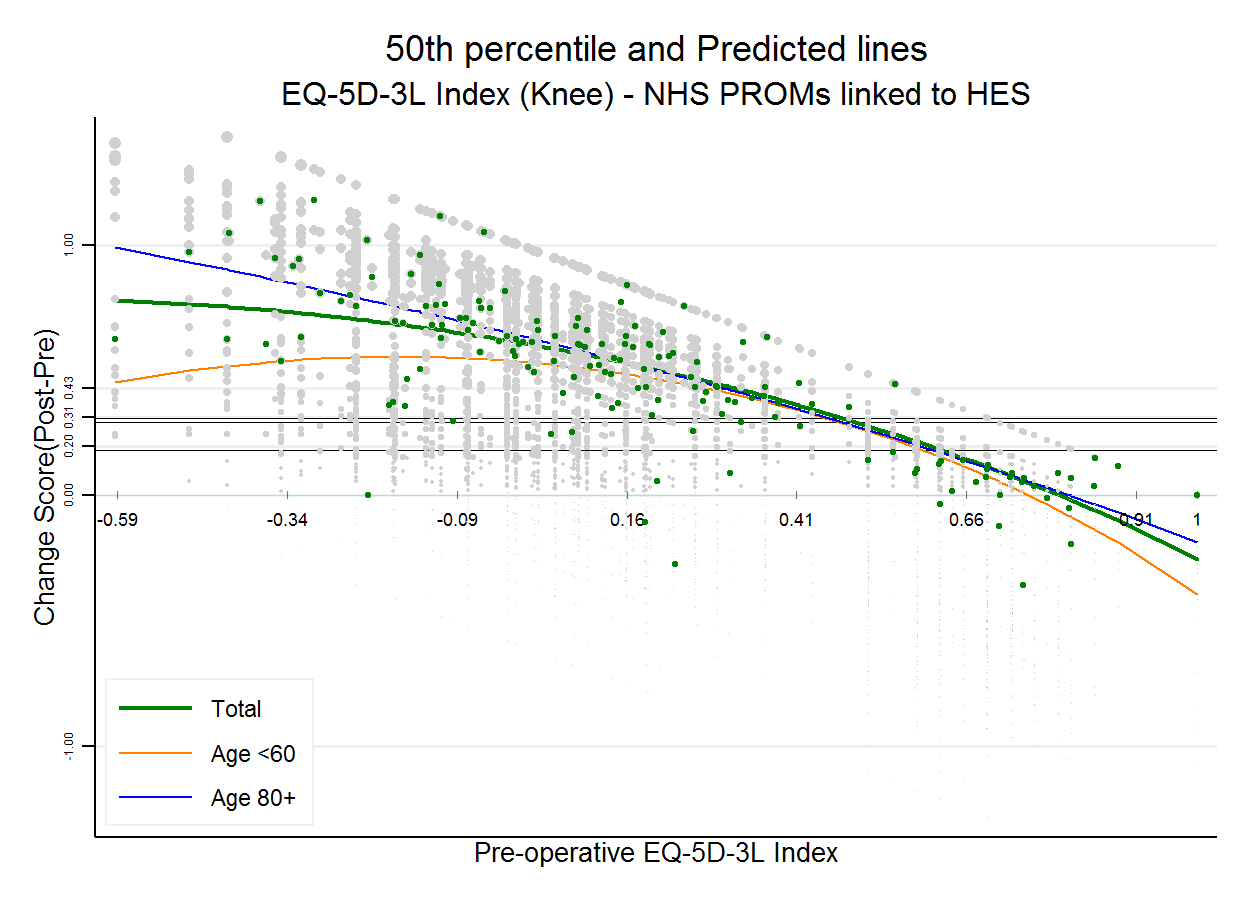

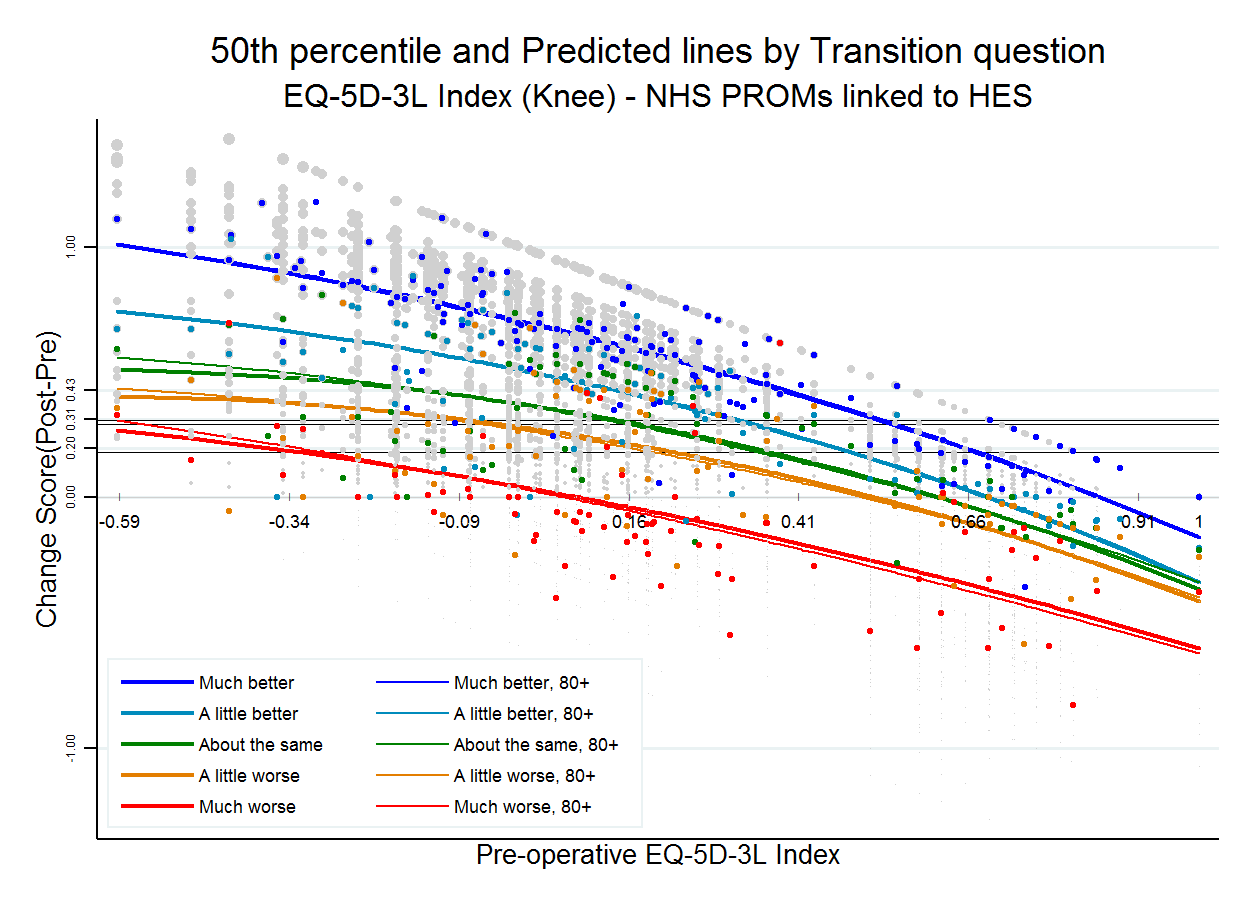


Supplementary Figure 1. The OKS and EQ-5D-3L - Total population (1, 3) and the transition question (2, 4)

Note: Fitted 3^rd^ degree Box-Cox regression lines (1) for the OKS total population and (2) by the patients’ self-assessment transition level. The 2^nd^ degree Box-Cox regression estimates (3) for the EQ-5D-3L total knee surgery population and (4) by the patients’ self-assessment transition level. All the graphs are presented by age group additionally.
Colourful dots indicate 50^th^ percentile for each category, and grey dots indicate actual observations.
Grey horizontal lines indicate each defined score improvement (e.g., 16 for the OKS and 0.309 for the hip EQ-5D-3L).
Percentiles of the EQ-5D-3L show all over disperse patterns by transition levels whereas percentiles of the OKS show disperse patterns in *‘A little worse’* and *‘Much worse’* transition level.

***Internal validation***

1 2

*
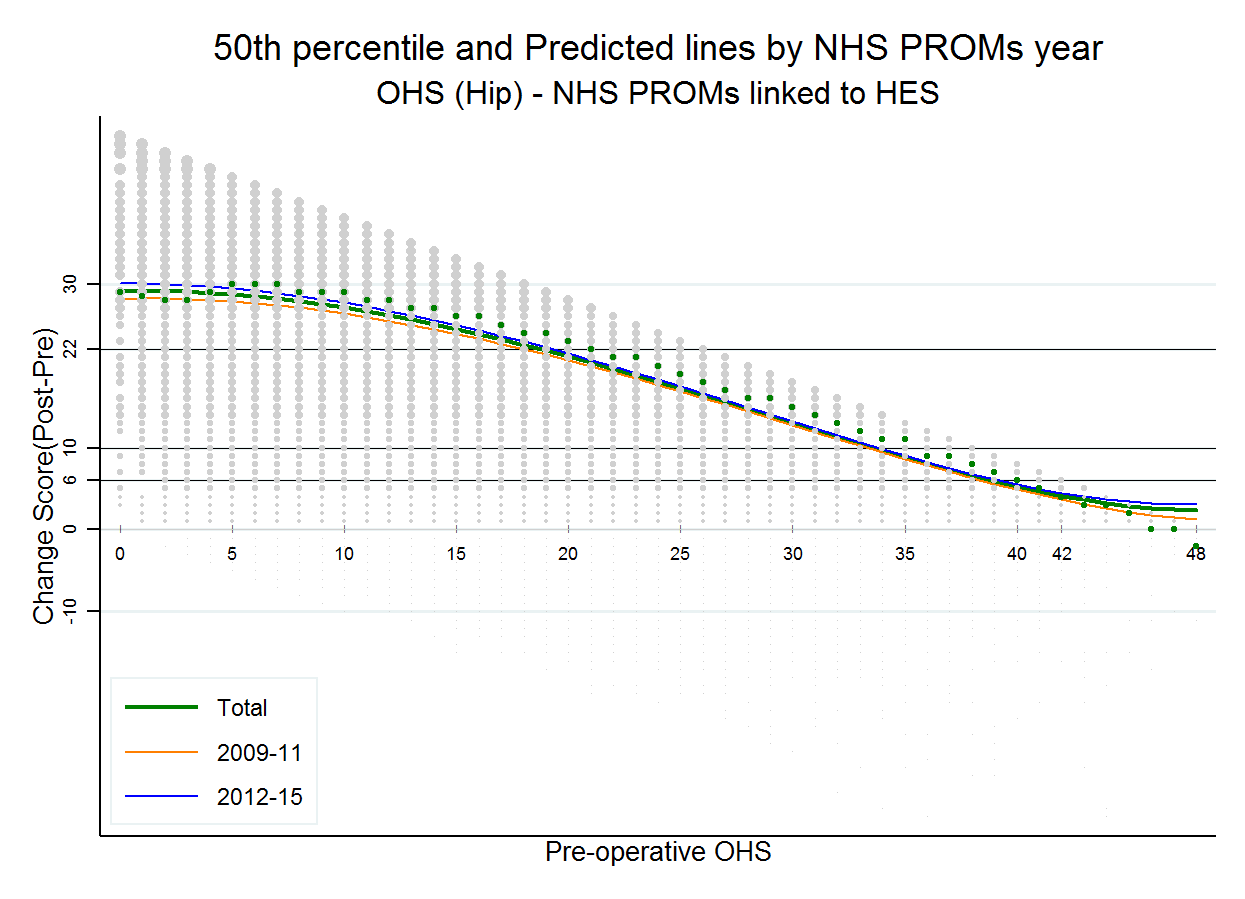

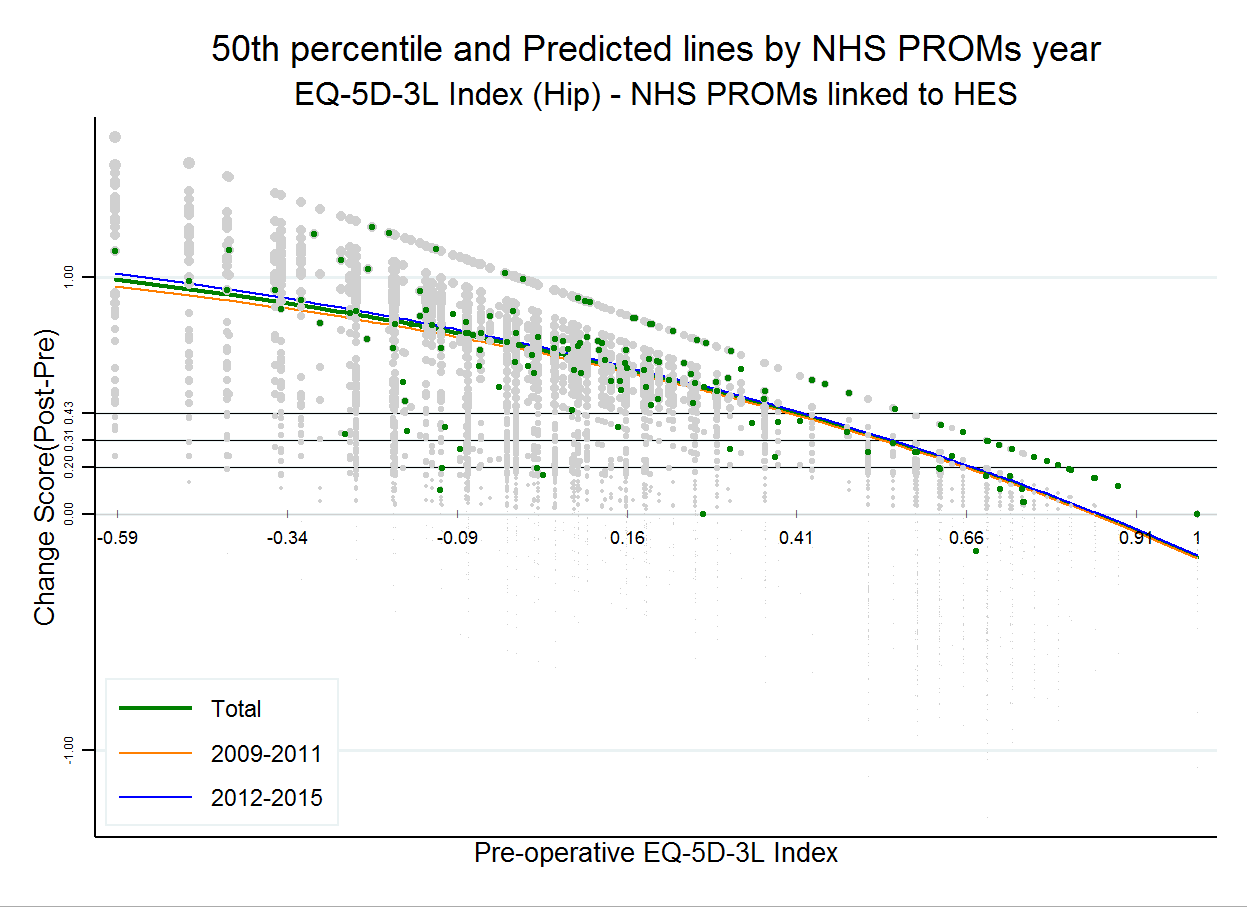
*

3 4


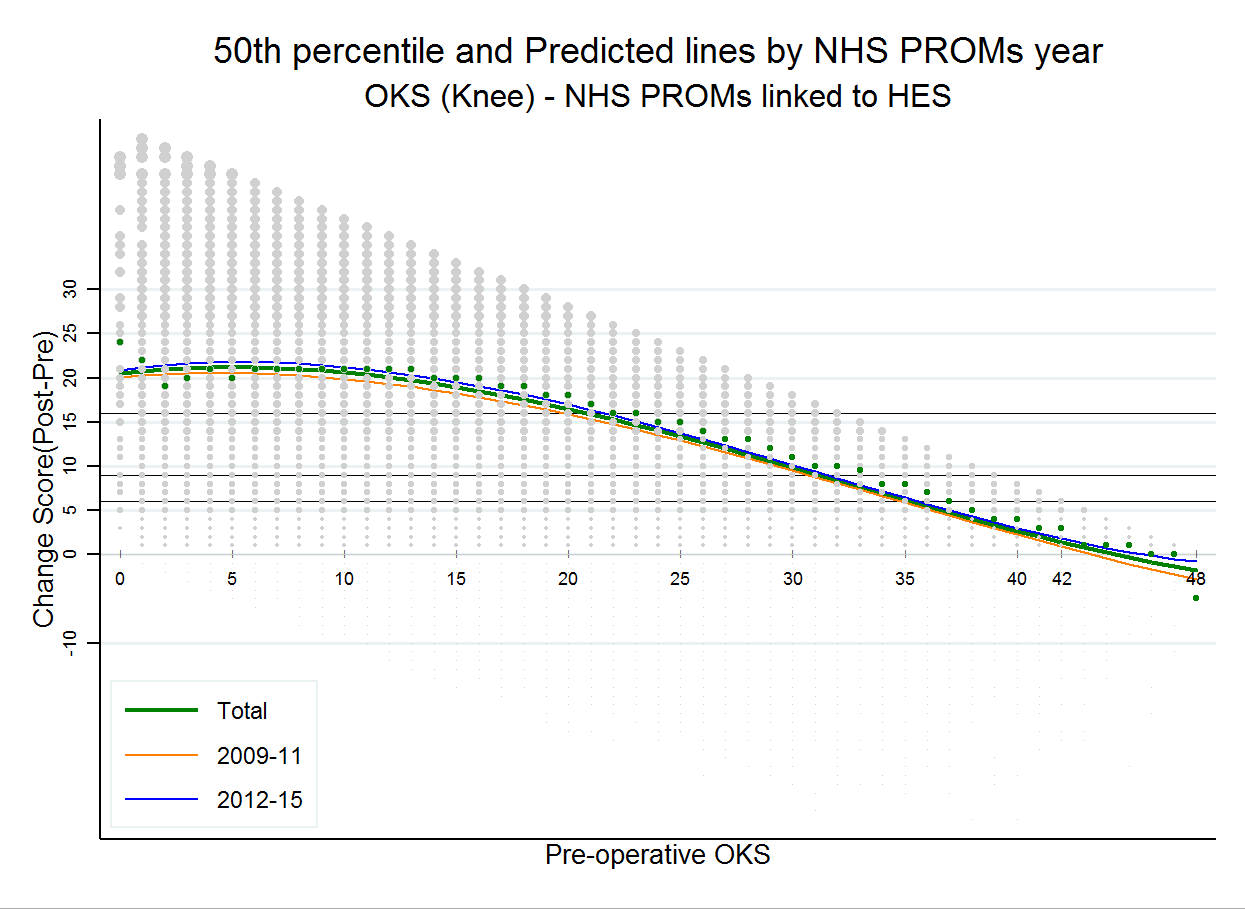

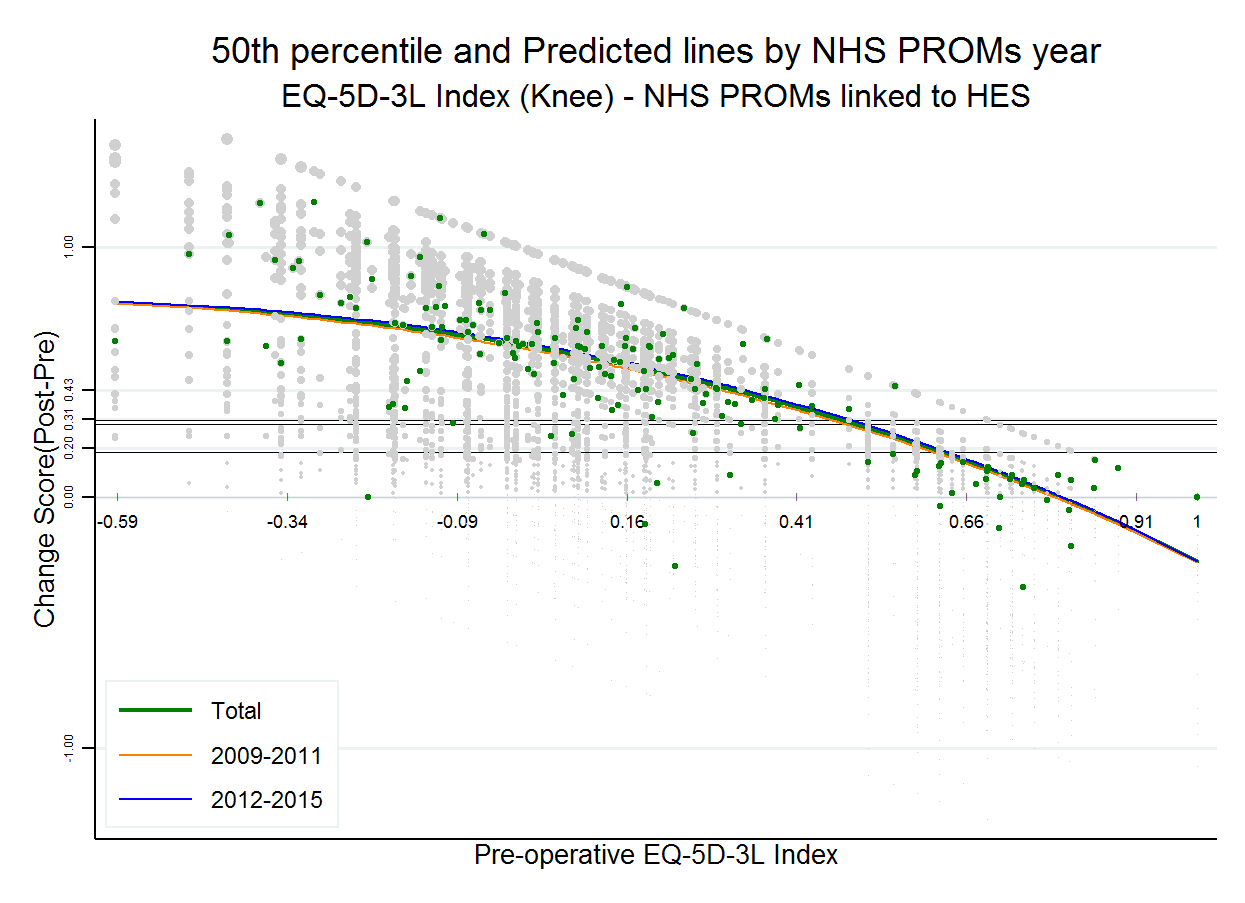


Supplementary Figure 2. The hip (1, 2) and the knee (3, 4) - Total population with NHS PROMs year

1 2

3 4

Supplementary Figure 3. Histograms of the OHS and the OKS changes (1, 3). Histograms of the EQ-5D-3L changes showed multimodal distributions (2, 4).

1 2

3 4

5 6

7 8

9 10

Supplementary Figure 4. The OHS (1, 3, 5, 7, 9) and the OKS (2, 4, 6, 8, 10) – Proportion and probabilities of improvement (using M MCID) by the transition question, using the 4^th^ degree Fractional polynomial logistic regressions.
